# Structural Comparison, Substrate Specificity, and Inhibitor Binding of AGPase Small Subunit from Monocot and Dicot: Present Insight and Future Potential

**DOI:** 10.1155/2014/583606

**Published:** 2014-09-02

**Authors:** Kishore Sarma, Priyabrata Sen, Madhumita Barooah, Manabendra D. Choudhury, Shubhadeep Roychoudhury, Mahendra K. Modi

**Affiliations:** ^1^Agri-Bioinformatics Promotion Programme, Department of Agricultural Biotechnology, Assam Agricultural University, Jorhat, Assam 785013, India; ^2^Department of Life Science & Bioinformatics, Assam University, Silchar, Assam 788011, India

## Abstract

ADP-glucose pyrophosphorylase (AGPase) is the first rate limiting enzyme of starch biosynthesis pathway and has been exploited as the target for greater starch yield in several plants. The structure-function analysis and substrate binding specificity of AGPase have provided enormous potential for understanding the role of specific amino acid or motifs responsible for allosteric regulation and catalytic mechanisms, which facilitate the engineering of AGPases. We report the three-dimensional structure, substrate, and inhibitor binding specificity of AGPase small subunit from different monocot and dicot crop plants. Both monocot and dicot subunits were found to exploit similar interactions with the substrate and inhibitor molecule as in the case of their closest homologue potato tuber AGPase small subunit. Comparative sequence and structural analysis followed by molecular docking and electrostatic surface potential analysis reveal that rearrangements of secondary structure elements, substrate, and inhibitor binding residues are strongly conserved and follow common folding pattern and orientation within monocot and dicot displaying a similar mode of allosteric regulation and catalytic mechanism. The results from this study along with site-directed mutagenesis complemented by molecular dynamics simulation will shed more light on increasing the starch content of crop plants to ensure the food security worldwide.

## 1. Introduction

**Figure 1:**
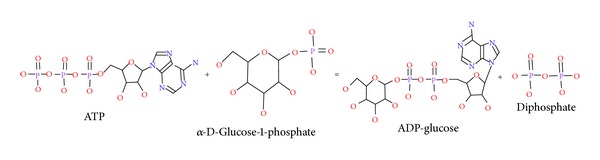


Starch is a basic constituent of the human and animal diet. It is an important carbohydrate considered as one of the primary energy sources for plants and a very important raw material for industrial processes. In many different plant species it has been demonstrated that ADP-glucose pyrophosphorylase (AGPase) (EC 2.7.7.27) is one of the major enzymes for starch biosynthesis. The overall crop yield potential is greatly influenced by the enzyme which modulates the photosynthetic efficiency in source tissues and determines the level of starch storage in sink tissues [1]. Combined participation of AGPase, starch synthase, and branching enzyme is solely responsible for biosynthesis of starch in plant [2, 3]. In starch biosynthesis, AGPase is the first regulatory allosteric enzyme which converts ATP and glucose-1-phosphate (Glc1P) to adenosine-5′-diphosphoglucose (ADPGlc) and inorganic pyrophosphate (PPi) [4–8] (see Figure 1).

Mutant analysis and transgenic plant provide strong evidences of the allosteric properties of AGPase in controlling the rate of starch biosynthesis in higher plants [9–13]. In most cases the regulation of AGPase depends on the ratio of 3-phosphoglyceric acid and inorganic phosphate (3PGA/Pi) showing a direct correlation between the concentration of 3-PGA and starch accumulation and an inverse correlation between Pi concentration and the starch content [14]. Although the overall kinetic mechanism of AGPase appears to be similar in bacteria and higher plants, their quaternary structures differ from each other [3]. Bacterial AGPases are composed of four identical subunits (α) to form α4 homotetramer whereas plant AGPases are heterotetramer of two different yet evolutionarily related subunits containing a pair of identical small (SS or α) and identical large subunits (LS or *β*) to form α2*β*2 heterotetramer [14–17]. The two subunits vary in their molecular weight and genetic origin and are encoded by two different genes [16, 18]. Primary sequence analysis of LS and SS of AGPase has shown considerable sequence homology, suggesting a common evolutionary origin [18]. Several researchers have reported that SS of AGPase has both catalytic and regulatory functions whereas LS has only regulatory function [19–24]. The hypothesis is well supported by former reports, reflecting SS is capable of forming a homotetramer with catalytic properties whereas LS is incompetent of forming an oligomeric structure with catalytic activities [1, 14, 20, 25]. In contrast, Kavakli et al. 2001 [26] and Hwang et al. 2006 and 2008 [27, 28] suggested that the LS may bind to substrate ATP as well as glucose-1 phosphate and may allow the LS to interact in tandem with the catalytic SS influencing the net catalysis. In addition, specific regions of both the LS and the SS were found to be important for enzyme stability and subunit association [1]. Study on chimeric maize/potato small subunits reflects a polymorphic motif of 55-amino acid region between the residues 322–376 plays a critical role during the interaction with LS and contributes to the overall stability of the enzyme [29]. All these reports suggest that both the subunits are of equal importance for the catalysis and allosteric regulation of the enzyme.

Due to the difficulty of obtaining AGPase in stable form neither the LS nor the heterotetrameric AGPase (α2*β*2) atomic resolution structure from plant species has been solved yet. In 2005 Jin et al. [30] reported the first atomic resolution structure of AGPase SS from* Solanum tuberosum*. The crystal structure of SS was found in a homotetrameric form. Since then not a single crystallographic structure of AGPase has been reported. Although the AGPase gene(s) offers an attractive tool for engineering crop plants to enhance the yield potential of starch content, the understanding of structure-function relationships and the unique substrate specificity of AGPase has remained elusive.

In the absence of experimental three-dimensional structures, comparative modeling of protein is considered as one of the most accurate methods of model building and is often considered fundamental for understanding their function [31]. This approach provides reasonable result based on the assumption that the tertiary structure of two proteins will be similar if they share high percentage of sequence similarity [32]. It is widely being used when there is a clear relationship of homology between the target protein sequences and at least with an experimental (XRD or NMR) protein structure. Comparative structural analysis coupled with docking study has been immensely used for understanding the structure function relationship, mode of enzyme substrate interaction, and key residues involved in interaction without requiring further biochemical or immunological data [33–48]. Comparative modeling of AGPase has previously been studied for understanding the structure function relationship and to investigate the subunit interaction for enzyme activity [28, 49–58].

In the present study, we report a comparative structure analysis of AGPase SS from different monocot and dicot crop plants based on the available atomic resolution structure of* S. tuberosum* AGPase SS (PDB ID: 1YP2). A detailed structural comparison of both monocot and dicot AGPase SS along with their specificity towards substrate (ATP) and inhibitor (sulphate) binding has been elucidated. The mode of interactions of the SS of AGPases with sulphate inhibitor is studied with the aid of molecular docking. Detailed structural comparison of AGPase SS and the key amino acid residues involved in substrate and inhibitor binding from the selected crop species will highlight the important structural aspects of AGPase SS and may provide insights into the enzyme's catalytic mechanism and understanding of the inhibitor binding specificity.

## 2. Materials and Methods

### 2.1. Computational Resources

All steps in this research were carried computationally on a Xeon, 2.13 GHz server equipped with the windows server 2003 environment. Preparation of three-dimensional structures, structure refinement, superimpositions, and docking were performed in Discovery Studio (DS3.5) (Accelrys, San Diego, CA, USA).

### 2.2. Sequence Analysis

Fasta formatted amino acid sequence of AGPase SS from three monocot crop plants, that is,* Oryza sativa *ssp*. japonica*,* Hordeum vulgare,* and* Triticum aestivum,* and six dicot crop plants, that is,* Arabidopsis thaliana*,* Solanum lycopersicum*,* Beta vulgaris*,* Vicia faba*,* Cicer arietinum,* and* Brassica napus,* was retrieved from the UniProtKB (http://www.uniprot.org/help/uniprotkb) database of ExPaSy. Primary structural study of the protein was done by computing various Physicochemical properties such as molecular weight, isoelectric point, instability index, aliphatic index, and grand average hydropathy (GRAVY) using ProtParam tool (http://web.expasy.org/protparam/) [59]. The secondary structure of AGPase SS was predicted from its primary amino acid sequence using CONCORD (http://helios.princeton.edu/CONCORD) [60] secondary structure prediction server. This is an accurate secondary structure prediction method that incorporates seven popular secondary structure prediction methods,* namely*, PSIPRED, DSC, GOR, Predator, Prof, PROFphd, and SSpro, for predicting the consensus out of them. The disordered regions of AGPase SS were predicted by protein disordered metaprediction server (metaPrDOS) (http://prdos.hgc.jp/meta/) [61].

### 2.3. Domain Analysis and Linker Prediction

Protein domain boundaries and architecture knowledge is essential for understanding and characterising of protein function. Detection of protein domain and architecture in the absence of three-dimensional structure benefits many areas of protein science, such as protein engineering and protein structure prediction [62]. Putative conserved domain, family, and superfamily possessed by AGPase SS were predicted based on sequence similarity search with its closest orthologous family members. Different tools and databases,* namely,* InterProScan (http://www.ebi.ac.uk/Tools/pfa/iprscan/) [63], Proteins Families Database (Pfam) (http://pfam.sanger.ac.uk/) [64], NCBI Conserved Domains Database (NCBI-CDD) (http://www.ncbi.nlm.nih.gov/Structure/cdd/cdd.shtml) [65], and SMART (http://smart.embl-heidelberg.de/) [66] server, were used for performing the task.

Overall functionality and efficiency of multiple domain proteins are affected by linker sequences. Cooperation and interaction between domains are affected by linker sequences which are flexible in 3D space, nonglobular, unstructured, or low complexity segment [67]. The linker sequence joining the discrete domains of AGPase SS was inferred manually.

### 2.4. Multiple Sequence Alignment

ClustalW [68] was used to construct a multiple sequence alignment of monocot and dicot AGPase SS along with their closest structural homologue* S. tuberosum* AGPase SS to have a better knowledge on conservation and variation of different amino acids at sequence level. ESPript (Easy Sequencing in PostScript) (http://www.ipbs.fr/ESPript) [69] was used for rendering the result which facilitates the rapid visualisation of sequence alignment via PostScript output. It produces a synthesis of sequence and structural information by reading secondary structure files such as that created by the program DSSP [70].

### 2.5. Comparative Modeling

#### 2.5.1. Template Identification, Model Building, and Refinement

Full length amino acid sequences of both monocot and dicot AGPase SS were subjected to BLASTP analysis against PDB in order to find suitable templates for comparative modeling. Blosum-62 matrix was used with a default threshold *E*-value of 10 and inclusion threshold value of 0.005. Template was selected based on high level of sequence identity, query coverage, and alignment quality which promises a more reliable and good quality model. GeneSilico MetaServer (https://genesilico.pl/meta2) [71] which uses a consensus approach to predict the template for model building was also employed to have better confidence and to ensure the sensitivity and accuracy of template selection for AGPase SS.

Theoretical three-dimensional modeling of AGPase SS was built using create homology model module of DS3.5. Initially twenty different models for each protein were built and were ranked according to their normalized discrete optimized protein energy (DOPE) scores and the model with the lowest DOPE score was selected for further validation. Modrefiner server (http://zhanglab.ccmb.med.umich.edu/ModRefiner/) [72] which is a high resolution protein structure refinement algorithm at atomic level was used to refine the modeled 3D structure of AGPase SS of both monocot and dicot closer to their native state. To increase the compatibility score of each residue, the target models were further refined by loop modeling and side chain refinement using DS3.5 ab-initio loop prediction algorithm Looper [73] and ChiRotor [74] for refining protein side-chain conformations. Looper generates a set of low energy conformations for the specified loop region and ChiRotor systematically search side-chain conformation and scores based on their CHARMM [75] energy. Out of five different models generated by Looper and ChiRotor, the best model was selected based on the lowest DOPE score. Refined models were subjected to energy minimisation by DS3.5 with the minimisation protocol. The minimisation protocol employs the steepest descent and conjugate gradient methods of minimisation algorithms used with a generalized born implicit solvent model. Parameters of a distance-dependent dielectric constant = 1, nonbonded radius of 14 Å, CHARMM force field, spherical electrostatic cutoff, and the steepest descent algorithm were used to remove close van der Waals contacts for maximum steps of 2000 with 0.1 minimising RMS gradient.

### 2.6. Structural Assessment and Refinement

Generated models were tested for quality by both geometric and energetic means. PROCHECK [76], ERRAT [77], and VERIFY3D [78] tools, which are embedded in structure analysis and validation server (SAVES) (http://nihserver.mbi.ucla.edu/SAVES/), were used for validation of the modeled proteins. The PROCHECK provides an idea of the stereo chemical quality of the protein. It analyses the Ramachandran plot quality, peptide bond planarity, nonbonded interactions, main chain hydrogen bond energy, Cα chiralities, and overall *G* factor. ERRAT checks the overall quality factor of the protein and was used to check the statistics of nonbonded interactions between different atom types. VERIFY3D was used to access the compatibility of the atomic models with its own amino acid sequence. A high VERIFY3D profile score indicates the high quality of protein model. MetaMQAPII (https://genesilico.pl/toolkit/unimod?method=MetaMQAPII) [79] which uses the result of VERIFY3D, PROSA, BALA, ANOLEA, PROVE, TUNE, REFINER, and PROQRES was used to identify global and/or local accuracy in models. Furthermore, secondary structure, solvent accessibility, and depth within the structure are also analysed by MetaMQAPII and are being used to assess the deviations of C-α atoms for a given model together with linear regression. Then, the global accuracy of the model is calculated based on these predictions and expressed as Global Distance Test_Total Score (GDT_TS). This indicator uses a set of distance thresholds of 1 Å, 2 Å, 4 Å, and 8 Å to find the average of the percentages of matched residue pairs between the C-α atoms in the model and the experimentally determined structure.

After each loop and side chain refinement steps, the above model quality assessment programs were employed to check the error at each residue in the protein. This process was repeated iteratively until the most geometrically and energetically stable structural conformation was attained.

To investigate how well the modeled structure matches the X-ray data of template protein, RMSD (root mean square deviation) between equivalent Cα and backbone atom pairs (target and template) was calculated by structural superimposition using superimpose module of DS3.5. STRIDE (http://webclu.bio.wzw.tum.de/stride/) [80] server was used to distinguish secondary structural elements of the predicted three-dimensional models from their atomic coordinates.

#### 2.6.1. Calculation of Noncovalent Interactions

Noncovalent interactions are weak electromagnetic interactions between atoms or molecules and help in understanding many chemical and biological phenomena. These interactions are critical in maintaining the three-dimensional structure of protein and hold the key to understand the molecular basis of stability and functions of protein. Various noncovalent interactions such as hydrogen bonds, ionic interactions, hydrophobic interactions, disulphide bonds, aromatic-aromatic interactions, aromatic-sulphur interactions, and Cation-pi interactions of both monocot and dicot AGPase SS were calculated and compared with the template protein using protein interactions calculator (PIC) server [81]. PIC server computes various interactions as mentioned above within a protein upon submission of the three- dimensional coordinate set of the protein.

### 2.7. Docking

Interaction between enzyme and its substrate provides an accurate picture of the interacting amino acid residues between the substrate and the active site. Different binding site prediction methods were employed for finding the binding site amino acid residues of both monocot and dicot AGPase SS. Binding sites of these models were selected based on the ligand-binding pocket of the template. DS3.5 binding site prediction module was also employed to predict the binding sites amino acid residues and functional residues which identifies the binding sites based on eraser and flood-filling algorithm [82]. MetaPocket2.0 server (http://projects.biotec.tu-dresden.de/metapocket/) [83] was also used for predicting binding sites of these models. It employs a consensus method by combining the results of four different methods (LIGSITEcs, PASS, Q-SiteFinder, and SURFNET) to improve the prediction success rate. The potential ligand binding sites were generated using a probe radius of 5.0 Å and the binding site having highest *z*-score was considered for further investigation.

Previous study by Jin et al., 2005, [30] reported that sulphate molecule acts as an inhibitor of* S. tuberosum* AGPase and later Boehlein et al., 2010, [84] explored the sulphate ion binding sites within the SS homotetramer of* S. tuberosum* to probe the allosteric binding sites of the maize endosperm AGPase. In addition, sulphate shows its structural similarity to all known allosteric regulators of higher plant AGPases which contain one or more phosphate moieties. For these reasons, we are using sulphate as an inhibitor to probe the allosteric binding sites of both monocot and dicot AGPase SS.

For ligand-protein interaction, both protein and ligand molecules were optimized using the “prepare protein and ligands tool” of DS3.5 which adds hydrogen ions to the protein and adds charges and hydrogen and applies force field to the ligand based on the CHARMM force field.

In this study, CDOCKER [85] module of DS3.5 was used to carry out the docking analysis. It is a grid based docking which uses CHARMM molecular simulation program to dock ligands within the active site of receptors. Prepared ligand molecule was docked into the active site of both monocot and dicot AGPase SS to elucidate its binding affinity towards the inhibitor molecule which in turn provides an insight into the allosteric regulation of the protein. The binding affinity of the ligand molecule into the active site of the protein was calculated based on the consensus scoring scheme of CDOCKER ENERGY, CDOCKER_Interaction Energy, Ligscore1_Dreiding, LigScore2_Dreiding, PLP1, PLP2, Jain, PMF, and PMF4 implemented in the protein-ligand interaction module of DS3.5.

## 3. Results and Discussions

### 3.1. Sequence Analysis

Single gene encoding the AGPase SS from monocot (*Oryza sativa* ssp.* japonica, Hordeum vulgare,* and* Triticum aestivum*) and dicot (*Arabidopsis thaliana, Solanum lycopersicum, Beta vulgaris,* and* Brassica napus*) crop plants was selected for the present study. In addition, two different SS encoding genes of dicot crop plant* Vicia faba* (AGPP, AGPC) and* Cicer arietinum* (CagpS1, CagpS2) were also considered for the present investigation. AGPP and AGPC genes of* Vicia faba* will be termed as* Vicia faba1* and* Vicia faba2* and CagpS1 and CagpS2 genes of* Cicer arietinum* will be termed as* Cicer arietinum1* and* Cicer arietinum2,* respectively, for simplicity. The UniProtKB accession number, organism name, gene name, number of amino acids, subunit structure, and subcellular location of AGPase SS belonging to monocot and dicot crop plants are shown in Table 1.

Primary sequence analysis of AGPase SS signifies that molecular weight of the selected sequences falls between 52 and 57 KDa. Theoretical isoelectric point, low GRAVY indices, and high aliphatic index suggest that both monocot and dicot AGPase SS under consideration are slightly acidic in nature, possess high affinity towards water, and are thermostable at wide range of temperatures. Protein with instability index less than 40 is predicted as stable or else unstable [86]. Computed instability index suggests that except* Oryza sativa* (P15280)*, Solanum lycopersicum* (Q42882),* Vicia faba*2 (P52417), and* Cicer arietinum*1 (Q9AT06), all other AGPase SS sequences under consideration are stable proteins. Computed physicochemical properties of AGPase SS from monocot and dicot crop plants are reported in Table 2.

Comparison of the predicted secondary structure statistics of both monocot and dicot AGPase SS along with their structural homologue using CONCORD reveals that random coils dominated among secondary structure elements followed by strands and helices which are shown in Table 3.

Proteins often in their native states have regions with very flexible and unstable structures treated as disordered regions which are involved in many biological processes such as regulation, signaling, and cell cycle control [87, 88]. During the interaction with ligands, it is often observed that disordered regions transit to order where the flexibility of the region provides high specificity and low affinity towards multiple partners [89]. Results from metaPrDOS reveal that the N-terminal amyloplast target sequence of approximately 80 residues and approximately five residues at the end of C-terminal region of AGPase SS is falling in the disordered region. It is also observed that the disordered regions of AGPase SS are prone to charged residues, low sequence complexity regions, and residues involved in phosphorylation.

### 3.2. Domain Analysis and Linker Prediction

Results from different domain prediction tools confirm a consensus prediction. Both monocot and dicot SS of AGPase are composed of an N-terminal catalytic ADP-glucose pyrophosphorylase domain and a C-terminal left-handed parallel beta helix domain. The N-terminal catalytic domain is approximately 200 residues and is structurally similar to Rossmann fold, typically present in nucleotide-binding domains. The C-terminal domain is composed of approximately 105 residues and is involved in cooperative allosteric regulation and oligomerisation. Domain positions of both the domains in the sequence are shown in Table 3. Smart analysis reflects that none of the SS of AGPase under investigation possess any transmembrane domain and they appeared to be soluble proteins.

Discrete domains are often associated with multiple functions of protein where domains are connected by inter-domain linkers. They keep the domains apart and provide great extent of flexibility to move individually. This phenomenon is a part of their catalytic function. The linker regions of AGPase SS were manually delineated and the amino acids propensities and order in linkers were examined. The multiple sequence alignment of the linker region (data not shown) shows a high percentage of sequence identity between monocot and dicot AGPase SS. Polar charged and uncharged hydrophilic residues and nonpolar hydrophobic residues are in equal propensities in the linker region. The number of proline residues is not adequate for rigidity of the linker which keeps apart the discrete domains present in AGPase SS.

### 3.3. Multiple Sequence Alignment

**Figure 2:**
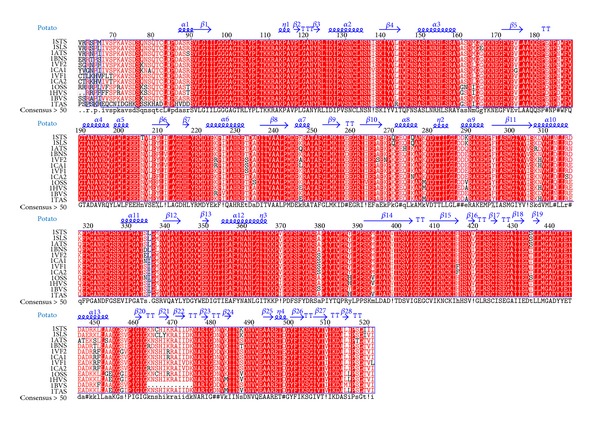
Multiple sequence alignment of AGPase SS from monocot and dicot crop plants using ClustalW. Conserved residues are highlighted in red. Secondary structural elements were imported from the crystal structure of* S. tuberosum* AGPase SS (PDB ID: 1YP2). The secondary structure and numbering are shown above the alignment in blue colour. α helices and *β* strands are represented with blue colour coil and arrow, respectively, and beta turns are marked with TT. 1STS, 1SLS, 1ATS, 1BNS, 1VF2, 1CA1, 1VF1, 1CA2, 1OSS, 1HVS, 1BVS, and 1TAS represent* Solanum tuberosum, Solanum lycopersicum, Arabidopsis thaliana, Brassica napus, Vicia faba2, Cicer arietinum1, Vicia faba1, Cicer arietinum2, Oryza sativa japonica, Hordeum vulgare, Beta vulgaris,* and* Triticum aestivum* AGPase SS, respectively.

The alignment shows a high percentage (more than 90%) of sequence identity between monocot and dicot crop plants under investigation. Secondary structure information of* S. tuberosum* AGPase SS was used for analysing the conservation of secondary structure elements which in turn provides the functional characteristics. Comparison of secondary structure elements (α helices and *β* strands) with* S. tuberosum* AGPase SS shows a high percentage of sequence conservation throughout the alignment which suggests a similar functionality of this protein in both monocot and dicot. Analysis of both the domain architecture and boundaries in the multiple alignments shows a high percentage of sequence conservation among all the AGPase SS along with their structural homologue. This reflects the conservation of domains throughout the evolutionary period and suggests their conserved role in substrate and inhibitor binding. The consensus sequence along with the template and the secondary structure elements is represented in Figure 2.

### 3.4. Comparative Modeling

BLAST search results reveal that high resolution (2.11 Ǻ) crystal structure of* S. tuberosum* AGPase SS (PDB ID: 1YP2_A) is the suitable template for model building. It has a high sequence identity, query coverage, less *E*-value, and a high level of alignment quality with query sequences.  Table 4 shows the query coverage, *E*-value, and sequence identity of the query sequences against their template protein* S. tuberosum* AGPase SS.

The crystal structure of* S. tuberosum* AGPase SS consists of 442 amino acids, lacking the amyloplast target sequence. Prior to modeling, the amyloplast target sequence of both monocot and dicot AGPase SS at the N-terminal end was removed to exclude the random coil fragment at this region and to achieve a better global superimposition with the template. Based on single-template approach, 20 different models were generated and the model having the lowest DOPE score is selected for model refinement and validation.

### 3.5. Model Quality Assessment

Different model quality assessment programs were employed for validating the refined and optimised models. The PROCHECK analysis shows that a high percentage of residue's *Ф* and Ψ angles are in the favoured region of Ramachandran plot contributing to the correctness of the models which is evident from Table 5 (see Supplementary Figure S1 available online at http://dx.doi.org/10.1155/2014/583606). Overall *G* factor is also in acceptable range as shown in Table 5 indicating that the designed models are of good quality and acceptable. An acceptable value of *G*-factor in Procheck is between 0 and −0.5 with the best model displaying values close to zero [90]. VERIFY3D program assessed the packing quality of each residue of the model where the compatibility of the model residues with their environment is assessed by a score function. Residues with a score over 0.2 (cut-off score > 0) should be considered reliable. It is evident from Table 5 that the score of both monocot and dicot models maximally lies above 0.2 which corresponds to acceptable side chain environment. A very high ERRAT score (Table 5) contributes to the acceptance of the models. Global distance total test score (GDT_TS)/RMSD score predicted by MetaMQAPII server is well above the cut-off score suggesting the acceptability of the models (Table 5). An ideal model has GDT_TS score over 59 and a RMSD around 2.0 Å. Model quality assessment score predicted by different programs is shown in Table 5.

The above model quality assessment programs check the stereo chemical quality, nonbonded interactions of the residues, the compatibility of the side chain environment, packing quality, and the energy profile of the predicted AGPase SS models and their result signify the high quality, reliability, and acceptability of the proposed models.

Computed RMSD of the Cα and backbone atom pairs for all the models are very low (Table 5). Low RMSD values indicate that the generated models are reasonably good and share high structural similarity and a common folding pattern with the template. Atomic coordinates of the models are deposited in Protein Model Database (PMDB) and can be accessed at http://mi.caspur.it/PMDB using PMDB ID: PM0079235-244 and PM0079249.

#### 3.5.1. Detailed Structural Comparison of AGPase Small Subunit

**Figure 3:**
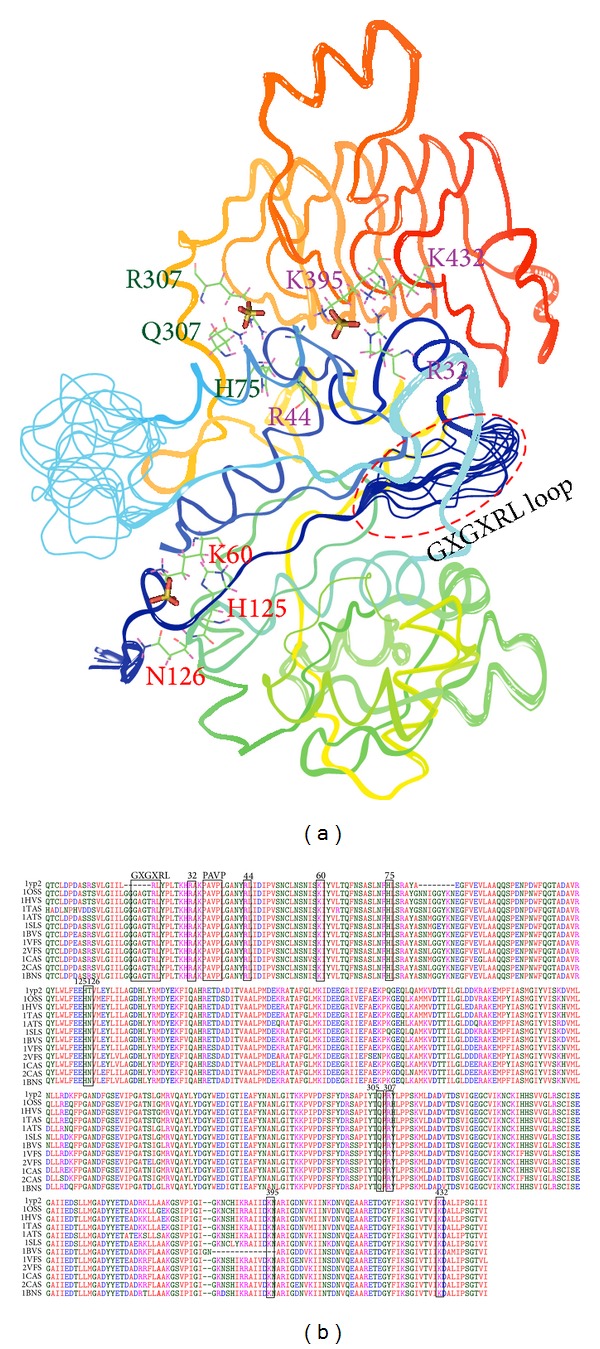
(a) Structural superimposition of both monocot and dicot AGPase SS along with the key residues responsible for allosteric binding. (b) Structural alignment showing the conservation of active site residues. Black boxes represent the active site residues along with their respective position. 1YP2, 1OSS, 1HVS, 1TAS, 1ATS, 1SLS, 1BVS, 1VFS, 2VFS, 1CAS, 2CAS, and 1BNS represent* Solanum tuberosum*,* Oryza sativa japonica*,* Hordeum vulgare*,* Triticum aestivum*,* Arabidopsis thaliana*,* Solanum lycopersicum*,* Beta vulgaris*,* Vicia faba*1,* Vicia faba*2,* Cicer arietinum*1,* Cicer arietinum*2, and* Brassica napus* AGPase SS, respectively.

Theoretical three-dimensional models of AGPases SS belonging to different monocot and dicot species were analysed extensively to have a wide spectrum on the three-dimensional structure and the role of key residues responsible for catalytic and inhibitory function. All the predicted structures of AGPase SS (Supplementary Figure S2) are composed of 15 helices (~23%), 10 3_10_ helices (2.3%), and 29-30 strands (~27.1%). Both monocot and dicot AGPase SS possess a Ψ loop and two gamma turns. The Ψ loop has a strand of 11 amino acid residues positioning Tyr217-Ser227 and a strand of 5 residues positioning Val260-Leu264 interconnected by a 32-residue long loop. The gamma loop forming residues of both monocot and dicot subunits are Gly40-Ala41-Asn42 and Val200-Asp201-Thr202. Structural comparison of modeled proteins shows strong conservation of secondary structural elements among them. Hydrogen bonding is most prominent among the beta turn forming residues thereby stabilising the connecting link of helices and strands. The comparative structural analysis of all the AGPase SS in the present investigation shares the same architecture. Structural superimposition of all the AGPase SS with its template shows that the secondary structure elements are superposed well and the key residues of allosteric regulation, that is, Arg32 equivalent to Arg41 in the template, Arg44/Arg53, Lys60/Lys69, His75/His84, His125/His134, Gln305/Gln314, Arg307/Arg316, Lys395/Lys404, and Lys432/Lys441, are fully conserved and are allosterically significant (Figure 3).

Tyrosine 135 which is a key residue within the active site of* S. tuberosum* AGPase SS for allosteric regulation was substituted to Asn126 in both monocot and dicot AGPase SS. Previous study on this enzyme reports that GXGXRL loop, PAVP motif, and residue equivalent to Arg33 in* S. tuberosum* SS play a key role for ATP binding which has been demonstrated by mutagenesis study [21, 91]. Structural superimposition shows that the strong conservation of GXGXRL loop positioning 20–25, PAVP motif positioning 35–38, and conservation of Arg24 (equivalent to Arg33 of* S. tuberosum* AGPase SS) in both monocot and dicot SS of AGPases firmly reflects the similar mode of action in this family of enzymes. Previous study by Jin et al. (2005) [30] concluded that metal-mediated catalytic mechanism is also used by AGPase. Residues equivalent to Asp145 and Asp280 (in* S. tuberosum* AGPase SS) chelate the metal ion and play a crucial role in metal-mediated catalytic mechanism. In several organisms the absolute need of a metal ion for AGPase has been biochemically demonstrated [6, 17, 92]. Taken together we have tried to check the binding specificity of these residues in our models to have a better confidence about the importance and involvement of these residues in metal-mediated catalytic mechanism across the family. Structural superimposition of our modeled proteins with the template protein reveals that Asp136 and Asp271 of both monocot and dicot equivalent to Asp145 and Asp280 of* S. tuberosum *AGPase SS are strongly conserved and follow common folding pattern with a very low RMSD difference which may prompt for metal-mediated catalytic mechanism. Comparison of secondary structure elements of AGPase SS from its three-dimensional coordinate using STRIDE (uses the three-dimensional structure for prediction of their secondary structure elements) (Table 6) reveals similar statistics to that of secondary structures from the primary amino acid sequence predicted by CONCORD server. This signifies the accuracy and reliability of the secondary structure elements of AGPase SS, assigned through homology modeling.

#### 3.5.2. Analysis on Noncovalent Interactions of Both Monocot and Dicot AGPase Small Subunit along with Their Structural Homologue

Collective effort of various noncovalent interactions determines the overall structure and behavior of proteins. An extensive hydrogen bond network (Main Chain-Main Chain/Main Chain-Side Chain/Side Chain-Side Chain) is observed in both monocot and dicot AGPase SS, which is parallel to the hydrogen bond network formed by their structural homologue. As protein folding and function is significantly contributed by hydrogen bonds, it is conceivable that both monocot and dicot AGPase SS share a common structural and functional similarity with that of the template protein. When positively charged amino acids and aromatic amino acids are in close proximity, they form the Cation-pi interactions which are significant to the protein structure. Arginine (R) is dominant over lysine (K) and the order of likeliness to participate in cation-pi interactions of aromatic amino acids is Tryptophan (W) followed by Tyrosine (Y) followed by Phenylalanine (F). In both monocot and dicot AGPase SS equal numbers of energetically significant cation-pi interactions were observed which is parallel to the cation-pi interactions of the template protein.

#### 3.5.3. Docking Analysis

**Figure 4:**
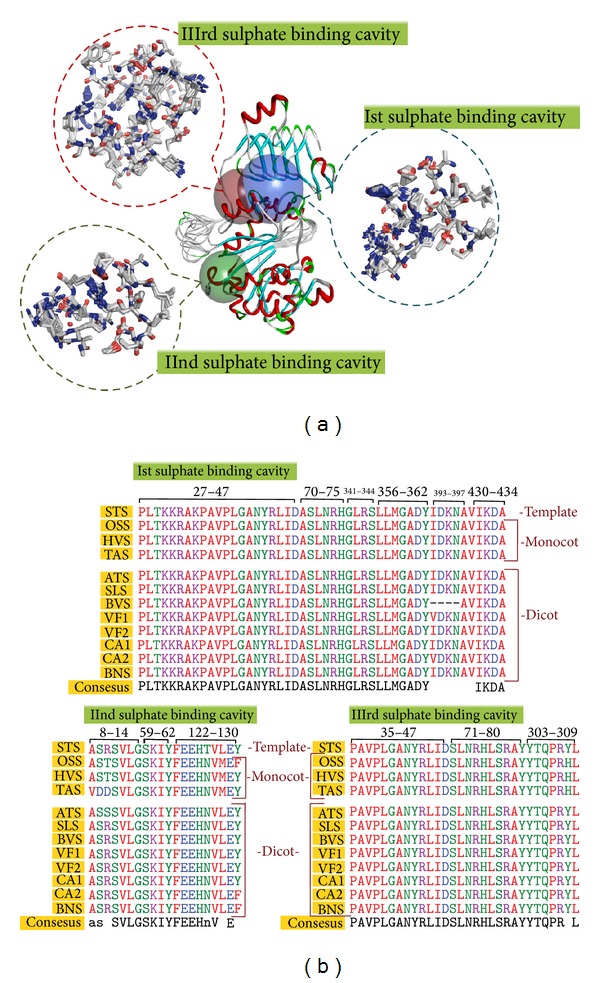
(a) Structural superimposition of both monocot and dicot AGPase SS structures with the template structure. Blue, green, and red solid spheres show the 1st, 2nd, and 3rd sulphate binding site of AGPase SS. Dotted circle represents the structural superimposition of active site residues in line representation. (b) Sequence alignment shows the 1st, 2nd, and 3rd sulphate binding cavity forming residues and their alignment. In the alignment STS, OSS, HVS, TAS, ATS, SLS, BVS, VF1, VF2, CA1, CA2, and BNS represent* Solanum tuberosum*,* Oryza sativa* ssp.* japonica*,* Hordeum vulgare*,* Triticum aestivum*,* Arabidopsis thaliana*,* Solanum lycopersicum*,* Beta vulgaris*,* Vicia faba*1,* Vicia faba*2,* Cicer arietinum*1,* Cicer arietinum*2, and* Brassica napus* AGPase SS, respectively.

**Figure 5:**
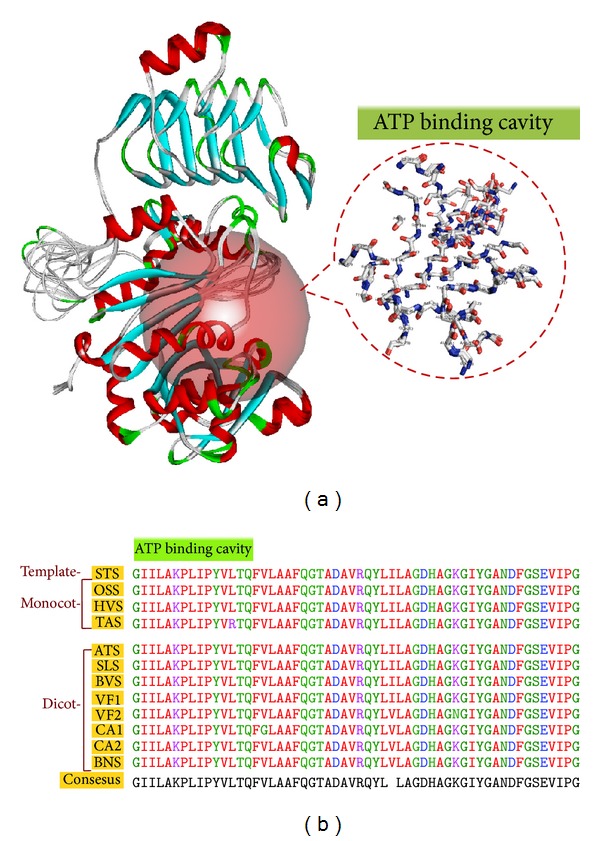
(a) Structural superimposition of both monocot and dicot AGPase SS structures with the template structure. Red solid sphere shows the ATP binding site of AGPase SS. Dotted circle represents the structural superimposition of ATP binding cavity forming residues in line representation. (b) Sequence alignment shows the ATP binding cavity forming residues in both monocot and dicot and their alignment. In the alignment STS, OSS, HVS, TAS, ATS, SLS, BVS, VF1, VF2, CA1, CA2, and BNS represent* Solanum tuberosum*,* Oryza sativa* ssp.* japonica*,* Hordeum vulgare*,* Triticum aestivum*,* Arabidopsis thaliana*,* Solanum lycopersicum*,* Beta vulgaris*,* Vicia faba*1,* Vicia faba*2,* Cicer arietinum*1,* Cicer arietinum*2, and* Brassica napus* AGPase SS, respectively. Sequence number of the participating residues is G(14)-L(17), A(33)-P(35), L(45), I(46), P(49), Y(62)-F(67), V(95)-A(98), F(108)-Y(118), L(131)-H(137), A(171), G(173), K(189), G(222)-Y(224), G(241)-P(251), and G(273).

The enzyme-substrate complex provides greater insight into the interactions and structural complementarities between the substrate and the active site of a protein. Assuming that the inhibitor binding modes are similar in the target and the template protein structure, active sites of the molecules were delineated based on the ligand-binding pocket of the template protein. The binding site module of DS3.5 and MetaPocket2.0 server also predicted the consensus binding site cavities and carries the similar conserved residues. Superposition (overlapping of Cα atoms) of modeled AGPase SS with their structural homologue in DS3.5 reveals that the spatial position and orientation of substrate and inhibitor binding site are highly conserved (Figures 4(a) and 4(b) and Figures 5(a) and 5(b)) and shows a very low RMSD within the substrate and inhibitor binding site which is evident from Table 7.

**Figure 6:**
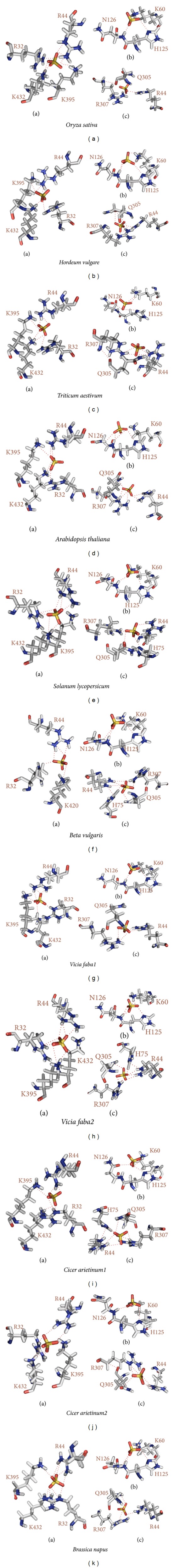
Docking of AGPase SS with sulphate inhibitor. (a), (b), and (c) represent the first, second, and third sulphate binding residues. Hydrogen bonds are represented with red dotted line and interacting residues are labeled.

Three sulphate molecules were successively docked into the active sites of both monocot and dicot AGPase SS to elucidate their structural and functional relevance in terms of inhibitor binding. Docking of inhibitor into the active site of modeled subunit reveals that Arg32, Arg44, Lys395, and Lys432 are directly involved in the interaction with the first sulphate molecule of both monocot and dicot AGPase SS by strong hydrogen and hydrophobic bonding, except for* Beta vulgaris* where Lys395 is missing. Tyr135 which is an aromatic, partially hydrophobic, amino acid was substituted to polar Asn126 in both monocot and dicot and plays a major role in the interaction with the second sulphate molecule along with Lys60 and His125 via hydrogen bonding. The third sulphate molecule binds with Arg44, Gln305, and Arg307 in both monocot and dicot AGPase SS, whereas H75 also shows its binding efficiency for sulphate in* Cicer arietinum*1,* Beta vulgaris*,* Solanum lycopersicum,* and* Vicia faba*2. Comparative docking study of sulphate inhibitor into the binding cavity of both monocot and dicot AGPase SS reflects a similar mode of binding specificity. Strong conservation and involvement of similar residues in the interaction convincingly suggest a similar inhibitor binding mechanism in both monocot and dicot AGPase SS in relation to the template protein. Stick representation of the docked complex of both monocot and dicot AGPase SS are shown in Figure 6.

#### 3.5.4. Comparison of Electrostatic Surface Potential of Binding Cavity from Both Monocot and Dicot AGPase Small Subunit

**Figure 7:**
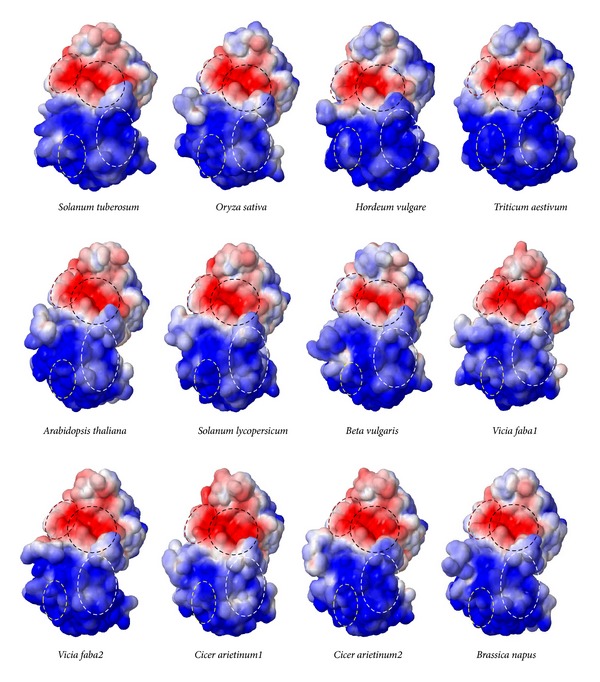
The electrostatic surface potential (generated with APBS) of monocot and dicot AGPase SS along with their structural homologue* S. tuberosum* AGPase SS is shown in Figure 7. Contour level for the electrostatic potential at the solvent-exposed surface is set to −5 (red) and +5 (blue) *kT*/*e*. Black, yellow, and red dotted circles represent the 1st, 2nd, and 3rd sulphate binding regions, respectively, and white dotted circle represents the ATP binding region.

The electrostatic surface potential energy of the modeled protein was calculated using the Adaptive Poisson-Boltzmann solver (APBS) package via PDB2PQR web portal (http://kryptonite.nbcr.net/pdb2pqr/) [93]. This state-of-the-art suite offers the computation of Poisson-Boltzmann electrostatic calculations on biomolecules. Energy refinement force field (AMBER) is used to assign atomic charges and ionic radii to the three- dimensional coordinate protein file. At pH 7.0, protonation state to the proteins is assigned by PROPKA. Solvent dielectric constant of 78.54 and a low dielectric constant of 2.0 were assigned to the protein. Most positively charged and most negatively charged surfaces were coloured using −5 and +5 *kT*/*e* settings where *k* is Boltzman Constant, *T* is temperature, and *e* is the charge of electron. Charge distribution and patches within the binding cavity of both monocot and dicot crop plants were studied extensively. Total electrostatic energies of both monocot and dicot AGPase SS range between 3.10 × 10^5^ kJ/mol and 3.18 × 10^5^ kJ/mol which is parallel to the total electrostatic energy of the template protein (3.12 × 10^5^ kJ/mol). The isocontour representation data fascinatingly displayed a similar topology with a similar electrostatic potential distribution pattern within the binding site of both monocot and dicot AGPase SS along with their template protein (Figure 7). This similar distribution of the electrostatic potential energy of both monocot and dicot AGPase SS substantiates similar structural organisation and perfectly correlates with their similar mode of binding activity.

## 4. Conclusions

The AGPase enzyme plays a pivotal role in starch biosynthesis for both photosynthetic and nonphotosynthetic plant tissues. Catalytic activity and allosteric regulation of this enzyme have significantly contributed to the overall yield potential of many crop plants. In the present study the theoretical three-dimensional models of AGPase SS from three different monocot and six different dicot crop plants were constructed for understanding the structure function relationship, substrate, and inhibitor binding specificity. The models were validated and further used for docking analysis with sulphate inhibitor. Superimposition of models with the crystal structure of the SS of* S. tuberosum* AGPase (PDB ID: 1YP2) shows a relatively low RMSD difference indicating a high structural similarity among the subunits. Structural superimposition of both monocot and dicot SS along with the template protein reflects strong conservation of secondary structure elements, common folding patterns, and similarity in the domains. GXGXRL loop and PAVP motif positioning 20–25 and 35–38, respectively, and Arg24 (equivalent to Arg33 of* S. tuberosum* AGPase SS) which play a crucial role for ATP binding are strongly conserved. It also follows a common folding pattern with a very low RMSD value in both monocot and dicot SS of AGPases reflecting similar mode of ATP binding in this family of enzymes. Structural superimposition of our modeled proteins with the template protein reveals that the aspartic acid positioning 136 and 271 (equivalent to Asp145 and Asp280 of* S. tuberosum*) of both monocot and dicot is strongly conserved and may act for metal mediated catalytic mechanism. Previous Alanine-scanning mutagenesis study performed by Boehlein et al., 2010, [84] suggests that mutating any of the Arg residues that are equivalent to* S. tuberosum* sulfate binding Arg residues drastically alters the maize AGPase overall allosteric properties. It clearly suggests the involvement of these Arg residues in allosteric effectors binding. Our docking study reveals the participation of similar Args (Arg32, Arg44, Arg307, and Arg361) residues in sulfate binding. Additionally Lys60, His75, His125, Asp126, Gln305, Lys395, and Lys432 are the key residues responsible for inhibitor binding and are allosterically significant in both monocot and dicot AGPase SS. The charge distribution patterns displayed a similar topology with a similar electrostatic potential distribution pattern within the binding site of both monocot and dicot AGPase SS and signify their similar mode of binding activity.

It is one of the major concerns about the analysis of the monocot AGPases because it is known that monocot plants like barley AGPases are insensitive to 3PGA and Pİ regulation. In addition to the barley endosperm AGPase, pea embryos and wheat grains and AGPases from nonphotosynthetic tissues have also been reported to be insensitive or weakly affected by 3PGA and Pİ regulation [94–96]. However, our findings are parallel with the result of Doan et al., 1999, [97] who suggested that barley endosperm AGPase SS is sensitive to 3-phosphoglycerate activation and is inhibited by inorganic phosphate although the active heterotetramer is allosterically unregulated. Therefore it can by hypothesised that SS of AGPases from both monocot and dicot under present investigation has similar substrate and inhibitor binding mechanism in this family of enzyme and the LS may be playing a crucial role for showing different variant of allosterically regulated and unregulated AGPases.

The active AGPase enzyme is a heterotetramer of two SS and two LS. Several studies have indicated that maximum stability, catalytic, and regulatory properties of the enzyme from higher plants result from the synergy of both SS and LS. Thus for understanding the actual mechanism of the active heterotetrameric AGPase and the dynamics involved in SS and LS assembly, their interaction and stability should be thoroughly studied. As the mode of action and dynamics of this protein depend on the delicate equilibrium between the protein and its native environment, further structural characterisation is bound to shed more light on the detailed mode of action of this family of protein in different crop species.

## Supplementary Material

Supplementary Figure S1. Ramachandran plot of both monocot and dicot AGPase small subunit. (i) Oryza sativa ssp. japonica AGPase small subunit, (ii) Hordeum vulgare AGPase small subunit, (iii) Triticum aestivum AGPase small subunit, (iv) Arabidopsis thaliana AGPase small subunit, (v) Solanum lycopersicum AGPase small subunit, (vi) Beta vulgaris AGPase small subunit, (vii) Vicia faba1 AGPase small subunit, (viii) Vicia faba2 AGPase small subunit, (ix) Cicer arietinum1 AGPase small subunit, (x) Cicer arietinum2 AGPase small subunit, (xi) Brassica napus AGPase small subunit .
Supplementary Figure S2. Theoritical three dimensional models of both monocot and dicot AGPase small subunit. (i) Oryza sativa ssp. japonica AGPase small subunit, (ii) Hordeum vulgare AGPase small subunit, (iii) Triticum aestivum AGPase small subunit, (iv) Arabidopsis thaliana AGPase small subunit, (v) Solanum lycopersicum AGPase small subunit, (vi) Beta vulgaris AGPase small subunit, (vii) Vicia faba1 AGPase small subunit, (viii) Vicia faba2 AGPase small subunit, (ix) Cicer arietinum1 AGPase small subunit, (x) Cicer arietinum2 AGPase small subunit, (xi) Brassica napus AGPase small subunit

## Figures and Tables

**Table 1:** SS of AGPase sequences analysed in this study.

UniProtKB accession number	Source (organism name)	Gene	Number of amino acids	Subunit structure	Subcellular location
Template (P23509)	*Solanum tuberosum *	AGPS	521	Heterotetramer	Chloroplastic/amyloplastic
MONOCOT					
P15280	*Oryza sativa* ssp. *japonica *	AGPS	514	Heterotetramer	Chloroplastic/amyloplastic
P55238	*Hordeum vulgare *		513	Heterotetramer	Chloroplastic/amyloplastic
P30523	*Triticum aestivum *	AGPS	473	Heterotetramer	Chloroplastic/amyloplastic
DICOT					
P55228	*Arabidopsis thaliana *	APS1	520	Heterotetramer	Chloroplastic
Q42882	*Solanum lycopersicum *		521	Heterotetramer	Chloroplastic
P55232	*Beta vulgaris *	AGPB1	489	Heterotetramer	Chloroplastic/amyloplastic
P52416	*Vicia faba*1	AGPC	508	Heterotetramer	Chloroplastic
P52417	*Vicia faba*2	AGPP	512	Heterotetramer	Chloroplastic
Q9AT06	*Cicer arietinum*1	CagpS1	516	Heterotetramer	Chloroplastic/amyloplastic
Q9AT05	*Cicer arietinum*2	CagpS2	505	Heterotetramer	Chloroplastic/amyloplastic
Q9M462	*Brassica napus *	AGPS1	520	Heterotetramer	Chloroplastic

**Table 2:** Physicochemical properties of AGPase SS.

AGPase	Molecular weight (Da)	Theoretical pI	Instability index	Aliphatic index	GRAVY
Template (*Solanum tuberosum*)	57240.3	6.73	44.18	91.21	−0.196
MONOCOT					
*Oryza sativa* ssp.* japonica *	56104.0	6.58	42.56	90.37	−0.159
*Hordeum vulgare *	56049.2	6.11	36.52	*91.72 *	*−0.118 *
*Triticum aestivum *	52417.4	5.54	35.91	90.76	−0.226
DICOT					
*Arabidopsis thaliana *	56650.5	6.13	34.86	93.04	−0.131
*Solanum lycopersicum *	57370.4	6.49	44.37	91.96	−0.201
*Beta vulgaris *	53796.2	5.59	38.06	91.19	−0.171
*Vicia faba*1	55627.4	6.43	37.94	91.79	−0.160
*Vicia faba*2	56059.6	6.19	40.56	90.14	−0.196
*Cicer arietinum*1	56104.0	6.58	42.56	90.37	−0.159
*Cicer arietinum*2	55309.9	6.20	34.80	91.19	−0.186
*Brassica napus *	57044.8	5.86	37.39	91.38	−0.215

**Table 3:** Secondary structure statistics of AGPase SS predicted by CONCORD and domain position.

AGPase	Helix (%)	Strand (%)	Coil (%)	Domain position
Template (*Solanum tuberosum*)	70 (13.44)	116 (22.26)	335 (64.30)	93–351, 389–515
MONOCOT				
*Oryza sativa *ssp. *japonica *	67 (13.03)	117 (22.76)	330 (64.20)	86–344, 382–507
*Hordeum vulgare *	68 (13.25)	113 (22.02)	332 (64.71)	85–343, 381–506
*Triticum aestivum *	66 (13.95)	112 (23.67)	295 (62.36)	45–303, 341–466
DICOT				
*Arabidopsis thaliana *	69 (13.26)	115 (22.11)	336 (64.61)	101–350, 388–514
*Solanum lycopersicum *	71 (13.62)	117 (22.45)	333 (63.91)	93–351, 389–515
*Beta vulgaris *	66 (13.49)	106 (21.67)	317 (64.82)	73–331, 369–483
*Vicia faba*1	71 (13.97)	118 (23.22)	319 (62.79)	80–338, 376–502
*Vicia faba*2	68 (13.28)	115 (22.46)	329 (64.25)	84–342, 380–506
*Cicer arietinum*1	70 (13.56)	116 (22.48)	330 (63.95)	88–346, 384–510
*Cicer arietinum*2	67 (13.26)	117 (23.16)	321 (63.56)	77–335, 373–499
*Brassica napus *	71 (13.65)	114 (21.92)	335 (64.42)	92–350, 388–514

**Table 4:** Query coverage, *E*-value, and sequence identity of the query sequences against *S. tuberosum* AGPase SS.

AGPase	Query coverage (%)	*E*-value	Sequence identity (%)	Template (PDB ID with chain identifier)
MONOCOT				
*Oryza sativa *ssp*. japonica *	87	0.0	92	1YP2_A
*Hordeum vulgare *	87	0.0	92	1YP2_A
*Triticum aestivum *	94	0.0	90	1YP2_A
DICOT				
*Arabidopsis thaliana *	86	0.0	94	1YP2_A
*Solanum lycopersicum *	86	0.0	99	1YP2_A
*Beta vulgaris *	89	0.0	92	1YP2_A
*Vicia faba*1	88	0.0	94	1YP2_A
*Vicia faba*2	87	0.0	93	1YP2_A
*Cicer arietinum*1	87	0.0	93	1YP2_A
*Cicer arietinum*2	89	0.0	94	1YP2_A
*Brassica napus *	86	0.0	95	1YP2_A

**Table 5:** Model quality assessment scores.

AGPase small subunit	Ramachandran plot (In %)				Overall *G*-factor	Errat	Verify 3D	MetaMQAP GDT/RMSD (Å)	RMSD (Å)
	Most favoured regions	Additional allowed regions	Generously allowed regions	Disallowed regions					
MONOCOT									
*Oryza sativa *ssp.* Japonica *	91.1	8.4	0.5	0.0	−0.06	77.60	98.65	83.880/1.919	C*α* = 0.25; backbone = 0.29
*Hordeum vulgare *	91.6	7.8	0.3	0.3	−0.06	82.03	98.65	84.615/1.537	Cα = 0.40; backbone = 0.42
*Triticum aestivum *	91.4	7.8	0.5	0.3	−0.10	79.72	100.00	84.842/1.583	Cα = 0.50; backbone = 0.51
DICOT									
*Arabidopsis thaliana *	90.4	9.1	0.3	0.3	−0.07	82.48	98.87	83.371/1.857	Cα = 0.28; backbone = 0.32
*Solanum lycopersicum *	92.5	7.3	0.0	0.3	−0.05	78.57	99.55	84.276/1.660	Cα = 0.23; backbone = 0.28
*Beta vulgaris *	92.2	7.0	0.5	0.3	−0.14	75.83	95.59	80.291/1.926	Cα = 0.45; backbone = 0.51
*Vicia faba*1	91.4	8.1	0.3	0.3	−0.06	85.48	97.74	83.088/1.757	Cα = 0.23; backbone = 0.29
*Vicia faba*2	91.1	8.3	0.0	0.5	−0.03	85.55	99.77	83.880/1.804	Cα = 0.19; backbone = 0.25
*Cicer arietinum*1	91.4	8.1	0.0	0.5	−0.05	82.16	97.07	83.201/1.778	Cα = 0.25; backbone = 0.31
*Cicer arietinum*2	91.2	8.6	0.0	0.3	−0.05	83.26	99.77	83.258/1.801	Cα = 0.21; backbone = 0.25
*Brassica napus *	90.1	8.8	0.5	0.5	−0.07	85.48	98.42	83.937/1.724	Cα = 0.31; backbone = 0.38

**Table 6:** Comparisons of secondary structure element of AGPase SS from its three dimensional coordinate.

AGPase	Alpha helix (%)	3–10 helix (%)	Strand (%)	Others (%)
MONOCOT				
*Oryza sativa *ssp.* japonica *	101 (22.9%)	10 (2.3%)	120 (27.1%)	211 (47.7%)
*Hordeum vulgare *	101 (22.9%)	10 (2.3%)	119 (26.9%)	212 (48.0%)
*Triticum aestivum *	101 (22.9%)	10 (2.3%)	119 (26.9%)	212 (48.0%)
DICOT				
*Arabidopsis thaliana *	101 (22.9%)	10 (2.3%)	119 (26.9%)	212 (48.0%)
*Solanum lycopersicum *	101 (22.9%)	10 (2.3%)	121 (27.4%)	210 (47.5%)
*Beta vulgaris *	101 (23.5%)	10 (2.3%)	113 (26.3%)	206 (47.9%)
*Vicia faba*1	101 (22.9%)	10 (2.3%)	122 (27.6%)	209 (47.3%)
*Vicia faba*2	101 (22.9%)	10 (2.3%)	121 (27.4%)	210 (47.5%)
*Cicer arietinum*1	101 (22.9%)	10 (2.3%)	119 (26.9%)	212 (48.0%)
*Cicer arietinum*2	101 (22.9%)	10 (2.3%)	119 (26.9%)	212 (48.0%)
*Brassica napus *	101 (22.9%)	10 (2.3%)	121 (27.4%)	210 (47.5%)

**Table 7:** RMSD comparisons of 1st, 2nd, and 3rd sulphate binding cavity and ATP binding cavity of both monocot and dicot AGPase SS.

AGPase	1st SO_4_ binding cavity (Å)	2nd SO_4_ binding cavity (Å)	3rd SO_4_ binding cavity (Å)	ATP binding cavity (Å)
MONOCOT				
*Oryza sativa *ssp. *japonica *	0.134	0.135	0.163	0.160
*Hordeum vulgare *	0.151	0.148	0.204	0.180
*Triticum aestivum *	0.164	0.207	0.191	0.190
DICOT				
*Arabidopsis thaliana *	0.155	0.180	0.200	0.149
*Solanum lycopersicum *	0.147	0.147	0.156	0.152
*Beta vulgaris *	0.212	0.161	0.171	0.175
*Vicia faba*1	0.142	0.166	0.172	0.147
*Vicia faba*2	0.136	0.173	0.169	0.156
*Cicer arietinum*1	0.153	0.171	0.171	0.139
*Cicer arietinum*2	0.137	0.157	0.177	0.157
*Brassica napus *	0.128	0.151	0.213	0.169
