# Supplementary figures and images for: Structural Comparison, Substrate Specificity, and Inhibitor Binding of AGPase Small Subunit from Monocot and Dicot: Present Insight and Future Potential
Source: Biomed Res Int. 2014 Sep 2;2014:583606. doi: 10.1155/2014/583606 (PMC4167649; doi:10.1155/2014/583606)

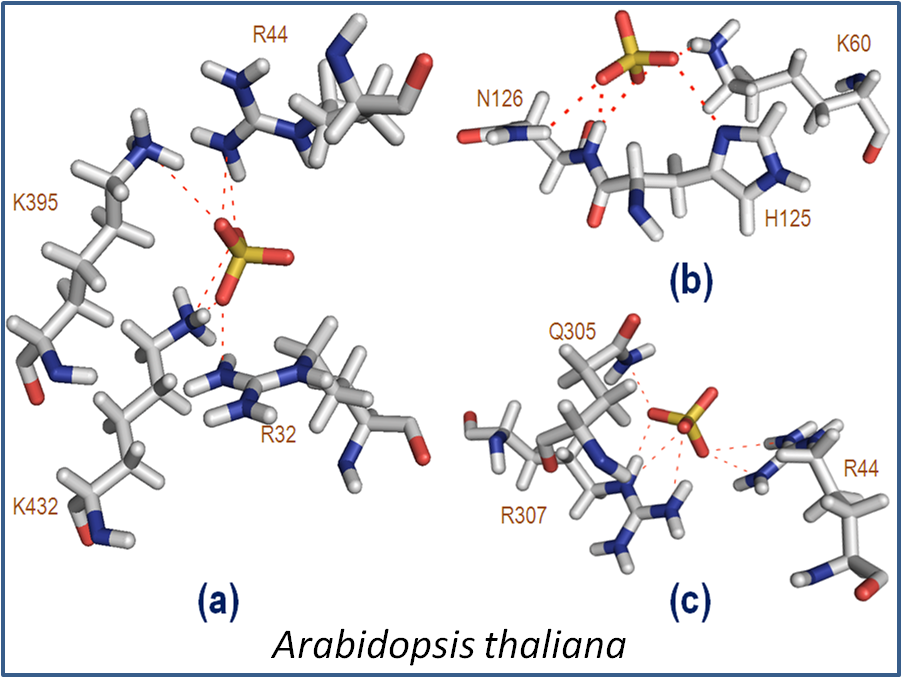

Supplement: Supplementary file 1 — Supplementary Figure S1. Ramachandran plot of both monocot and dicot AGPase small subunit. (i) Oryza sativa ssp. japonica AGPase small subunit, (ii) Hordeum vulgare AGPase small subunit, (iii) Triticum aestivum AGPase small subunit, (iv) Arabidopsis thaliana AGPase small subunit, (v) Solanum lycopersicum AGPase small subunit, (vi) Beta vulgaris AGPase small subunit, (vii) Vicia faba1 AGPase small subunit, (viii) Vicia faba2 AGPase small subunit, (ix) Cicer arietinum1 AGPase small subunit, (x) Cicer arietinum2 AGPase small subunit, (xi) Brassica napus AGPase small subunit . Supplementary Figure S2. Theoritical three dimensional models of both monocot and dicot AGPase small subunit. (i) Oryza sativa ssp. japonica AGPase small subunit, (ii) Hordeum vulgare AGPase small subunit, (iii) Triticum aestivum AGPase small subunit, (iv) Arabidopsis thaliana AGPase small subunit, (v) Solanum lycopersicum AGPase small subunit, (vi) Beta vulgaris AGPase small subunit, (vii) Vicia faba1 AGPase small subunit, (viii) Vicia faba2 AGPase small subunit, (ix) Cicer arietinum1 AGPase small subunit, (x) Cicer arietinum2 AGPase small subunit, (xi) Brassica napus AGPase small subunit [file 583606.f1.zip › supplementary/A. thaliana.TIF]

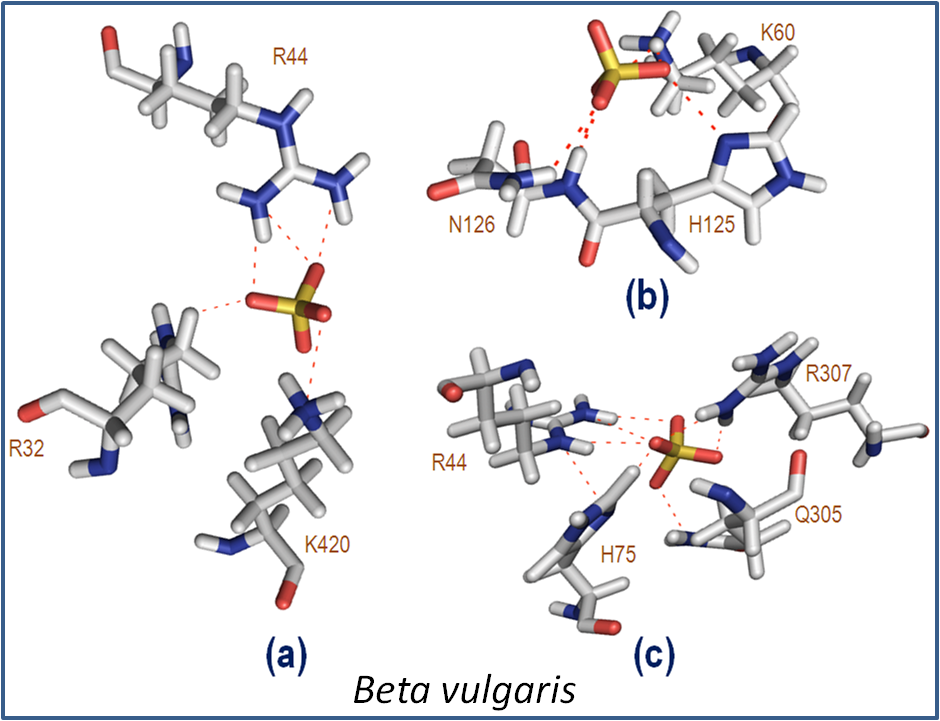

Supplement: Supplementary file 1 — Supplementary Figure S1. Ramachandran plot of both monocot and dicot AGPase small subunit. (i) Oryza sativa ssp. japonica AGPase small subunit, (ii) Hordeum vulgare AGPase small subunit, (iii) Triticum aestivum AGPase small subunit, (iv) Arabidopsis thaliana AGPase small subunit, (v) Solanum lycopersicum AGPase small subunit, (vi) Beta vulgaris AGPase small subunit, (vii) Vicia faba1 AGPase small subunit, (viii) Vicia faba2 AGPase small subunit, (ix) Cicer arietinum1 AGPase small subunit, (x) Cicer arietinum2 AGPase small subunit, (xi) Brassica napus AGPase small subunit . Supplementary Figure S2. Theoritical three dimensional models of both monocot and dicot AGPase small subunit. (i) Oryza sativa ssp. japonica AGPase small subunit, (ii) Hordeum vulgare AGPase small subunit, (iii) Triticum aestivum AGPase small subunit, (iv) Arabidopsis thaliana AGPase small subunit, (v) Solanum lycopersicum AGPase small subunit, (vi) Beta vulgaris AGPase small subunit, (vii) Vicia faba1 AGPase small subunit, (viii) Vicia faba2 AGPase small subunit, (ix) Cicer arietinum1 AGPase small subunit, (x) Cicer arietinum2 AGPase small subunit, (xi) Brassica napus AGPase small subunit [file 583606.f1.zip › supplementary/B. vulgaris.TIF]

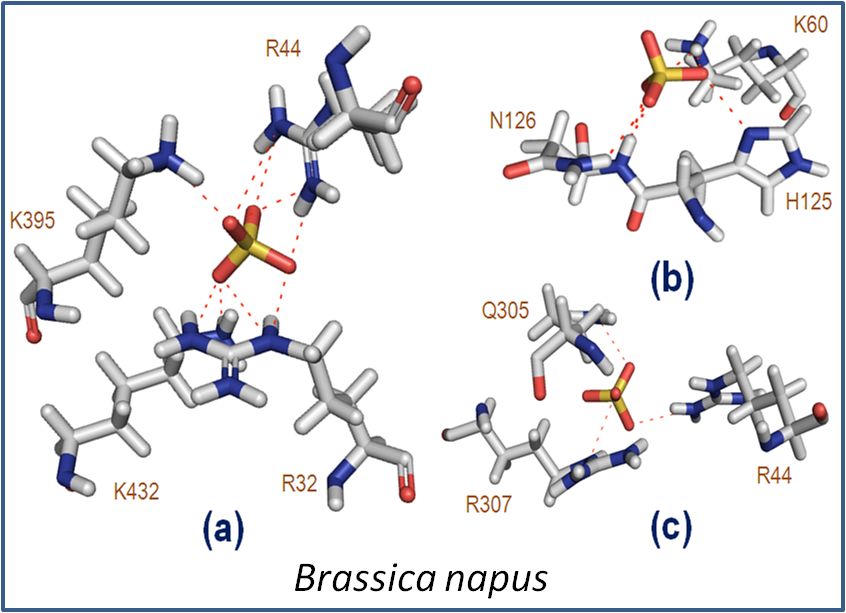

Supplement: Supplementary file 1 — Supplementary Figure S1. Ramachandran plot of both monocot and dicot AGPase small subunit. (i) Oryza sativa ssp. japonica AGPase small subunit, (ii) Hordeum vulgare AGPase small subunit, (iii) Triticum aestivum AGPase small subunit, (iv) Arabidopsis thaliana AGPase small subunit, (v) Solanum lycopersicum AGPase small subunit, (vi) Beta vulgaris AGPase small subunit, (vii) Vicia faba1 AGPase small subunit, (viii) Vicia faba2 AGPase small subunit, (ix) Cicer arietinum1 AGPase small subunit, (x) Cicer arietinum2 AGPase small subunit, (xi) Brassica napus AGPase small subunit . Supplementary Figure S2. Theoritical three dimensional models of both monocot and dicot AGPase small subunit. (i) Oryza sativa ssp. japonica AGPase small subunit, (ii) Hordeum vulgare AGPase small subunit, (iii) Triticum aestivum AGPase small subunit, (iv) Arabidopsis thaliana AGPase small subunit, (v) Solanum lycopersicum AGPase small subunit, (vi) Beta vulgaris AGPase small subunit, (vii) Vicia faba1 AGPase small subunit, (viii) Vicia faba2 AGPase small subunit, (ix) Cicer arietinum1 AGPase small subunit, (x) Cicer arietinum2 AGPase small subunit, (xi) Brassica napus AGPase small subunit [file 583606.f1.zip › supplementary/B.napus.TIF]

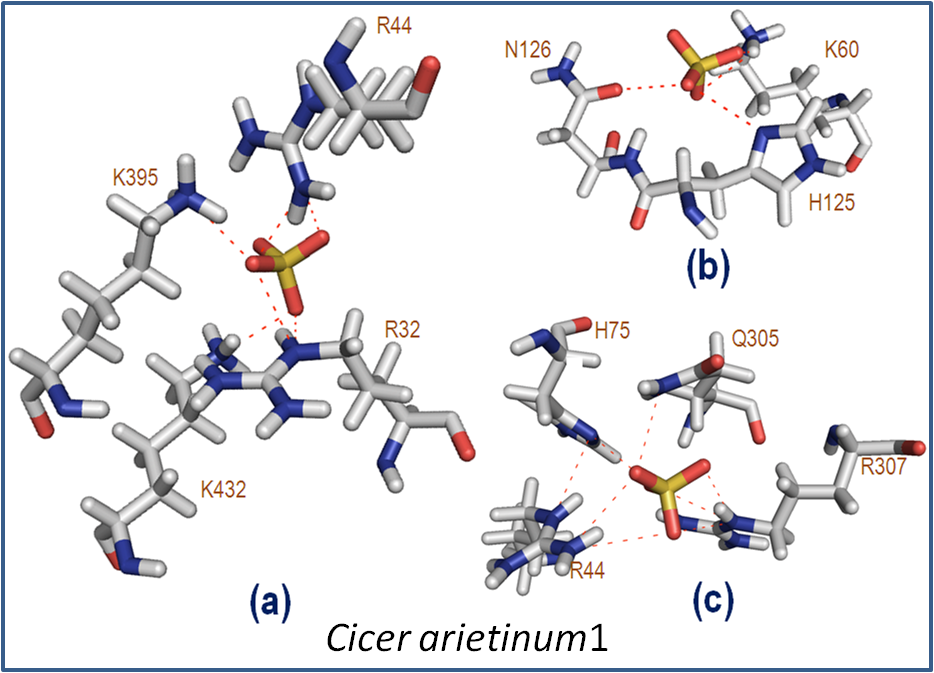

Supplement: Supplementary file 1 — Supplementary Figure S1. Ramachandran plot of both monocot and dicot AGPase small subunit. (i) Oryza sativa ssp. japonica AGPase small subunit, (ii) Hordeum vulgare AGPase small subunit, (iii) Triticum aestivum AGPase small subunit, (iv) Arabidopsis thaliana AGPase small subunit, (v) Solanum lycopersicum AGPase small subunit, (vi) Beta vulgaris AGPase small subunit, (vii) Vicia faba1 AGPase small subunit, (viii) Vicia faba2 AGPase small subunit, (ix) Cicer arietinum1 AGPase small subunit, (x) Cicer arietinum2 AGPase small subunit, (xi) Brassica napus AGPase small subunit . Supplementary Figure S2. Theoritical three dimensional models of both monocot and dicot AGPase small subunit. (i) Oryza sativa ssp. japonica AGPase small subunit, (ii) Hordeum vulgare AGPase small subunit, (iii) Triticum aestivum AGPase small subunit, (iv) Arabidopsis thaliana AGPase small subunit, (v) Solanum lycopersicum AGPase small subunit, (vi) Beta vulgaris AGPase small subunit, (vii) Vicia faba1 AGPase small subunit, (viii) Vicia faba2 AGPase small subunit, (ix) Cicer arietinum1 AGPase small subunit, (x) Cicer arietinum2 AGPase small subunit, (xi) Brassica napus AGPase small subunit [file 583606.f1.zip › supplementary/C. arietinum1.TIF]

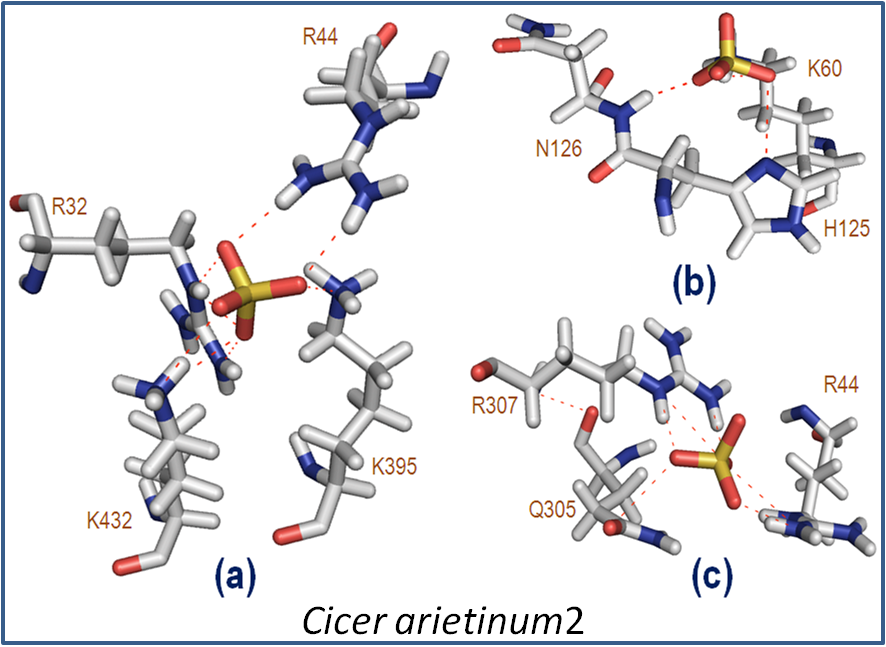

Supplement: Supplementary file 1 — Supplementary Figure S1. Ramachandran plot of both monocot and dicot AGPase small subunit. (i) Oryza sativa ssp. japonica AGPase small subunit, (ii) Hordeum vulgare AGPase small subunit, (iii) Triticum aestivum AGPase small subunit, (iv) Arabidopsis thaliana AGPase small subunit, (v) Solanum lycopersicum AGPase small subunit, (vi) Beta vulgaris AGPase small subunit, (vii) Vicia faba1 AGPase small subunit, (viii) Vicia faba2 AGPase small subunit, (ix) Cicer arietinum1 AGPase small subunit, (x) Cicer arietinum2 AGPase small subunit, (xi) Brassica napus AGPase small subunit . Supplementary Figure S2. Theoritical three dimensional models of both monocot and dicot AGPase small subunit. (i) Oryza sativa ssp. japonica AGPase small subunit, (ii) Hordeum vulgare AGPase small subunit, (iii) Triticum aestivum AGPase small subunit, (iv) Arabidopsis thaliana AGPase small subunit, (v) Solanum lycopersicum AGPase small subunit, (vi) Beta vulgaris AGPase small subunit, (vii) Vicia faba1 AGPase small subunit, (viii) Vicia faba2 AGPase small subunit, (ix) Cicer arietinum1 AGPase small subunit, (x) Cicer arietinum2 AGPase small subunit, (xi) Brassica napus AGPase small subunit [file 583606.f1.zip › supplementary/C. arietinum2.TIF]

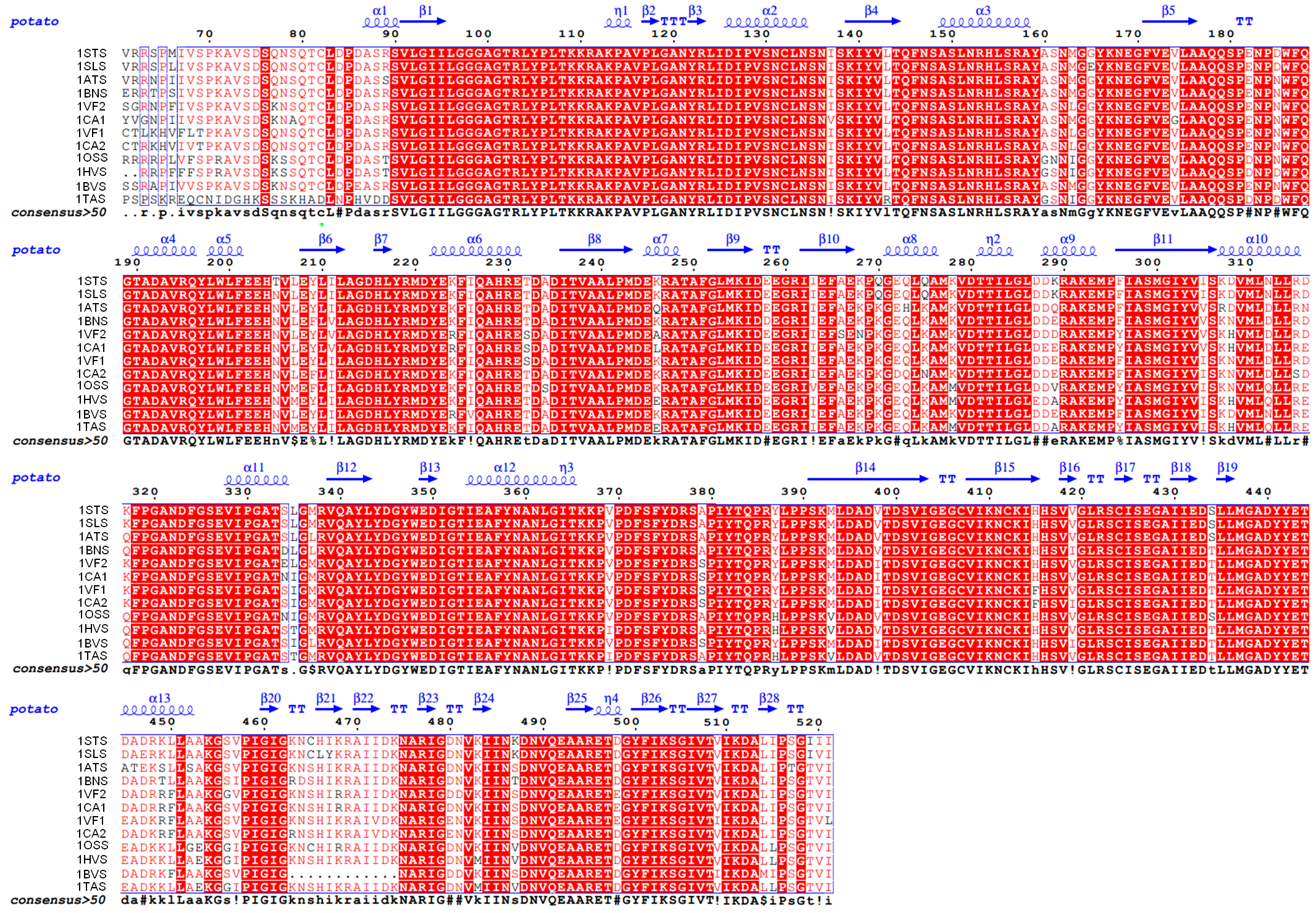

Supplement: Supplementary file 1 — Supplementary Figure S1. Ramachandran plot of both monocot and dicot AGPase small subunit. (i) Oryza sativa ssp. japonica AGPase small subunit, (ii) Hordeum vulgare AGPase small subunit, (iii) Triticum aestivum AGPase small subunit, (iv) Arabidopsis thaliana AGPase small subunit, (v) Solanum lycopersicum AGPase small subunit, (vi) Beta vulgaris AGPase small subunit, (vii) Vicia faba1 AGPase small subunit, (viii) Vicia faba2 AGPase small subunit, (ix) Cicer arietinum1 AGPase small subunit, (x) Cicer arietinum2 AGPase small subunit, (xi) Brassica napus AGPase small subunit . Supplementary Figure S2. Theoritical three dimensional models of both monocot and dicot AGPase small subunit. (i) Oryza sativa ssp. japonica AGPase small subunit, (ii) Hordeum vulgare AGPase small subunit, (iii) Triticum aestivum AGPase small subunit, (iv) Arabidopsis thaliana AGPase small subunit, (v) Solanum lycopersicum AGPase small subunit, (vi) Beta vulgaris AGPase small subunit, (vii) Vicia faba1 AGPase small subunit, (viii) Vicia faba2 AGPase small subunit, (ix) Cicer arietinum1 AGPase small subunit, (x) Cicer arietinum2 AGPase small subunit, (xi) Brassica napus AGPase small subunit [file 583606.f1.zip › supplementary/FIG.1.TIF]

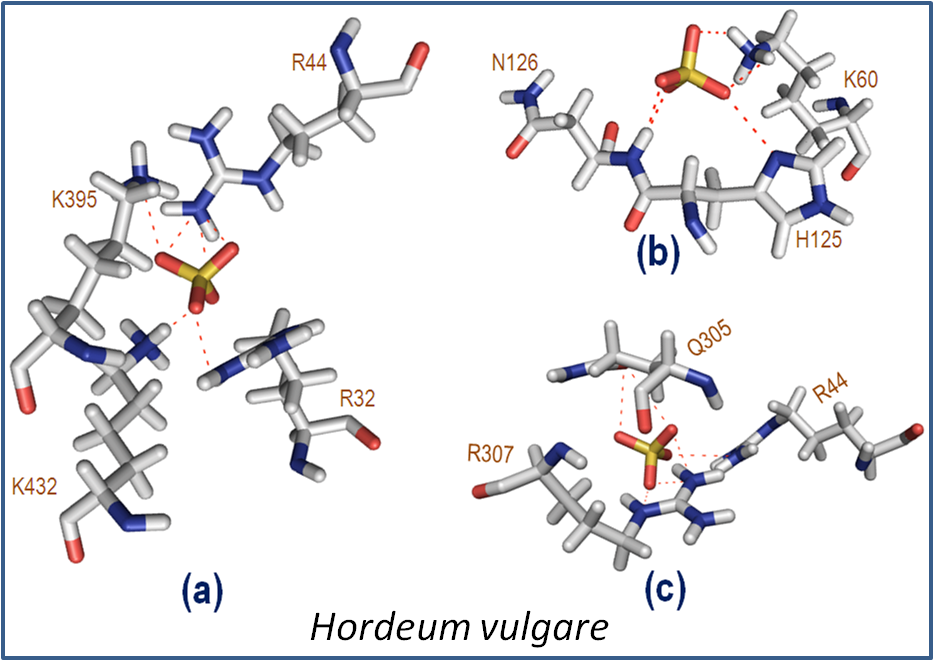

Supplement: Supplementary file 1 — Supplementary Figure S1. Ramachandran plot of both monocot and dicot AGPase small subunit. (i) Oryza sativa ssp. japonica AGPase small subunit, (ii) Hordeum vulgare AGPase small subunit, (iii) Triticum aestivum AGPase small subunit, (iv) Arabidopsis thaliana AGPase small subunit, (v) Solanum lycopersicum AGPase small subunit, (vi) Beta vulgaris AGPase small subunit, (vii) Vicia faba1 AGPase small subunit, (viii) Vicia faba2 AGPase small subunit, (ix) Cicer arietinum1 AGPase small subunit, (x) Cicer arietinum2 AGPase small subunit, (xi) Brassica napus AGPase small subunit . Supplementary Figure S2. Theoritical three dimensional models of both monocot and dicot AGPase small subunit. (i) Oryza sativa ssp. japonica AGPase small subunit, (ii) Hordeum vulgare AGPase small subunit, (iii) Triticum aestivum AGPase small subunit, (iv) Arabidopsis thaliana AGPase small subunit, (v) Solanum lycopersicum AGPase small subunit, (vi) Beta vulgaris AGPase small subunit, (vii) Vicia faba1 AGPase small subunit, (viii) Vicia faba2 AGPase small subunit, (ix) Cicer arietinum1 AGPase small subunit, (x) Cicer arietinum2 AGPase small subunit, (xi) Brassica napus AGPase small subunit [file 583606.f1.zip › supplementary/H. vulgare.TIF]

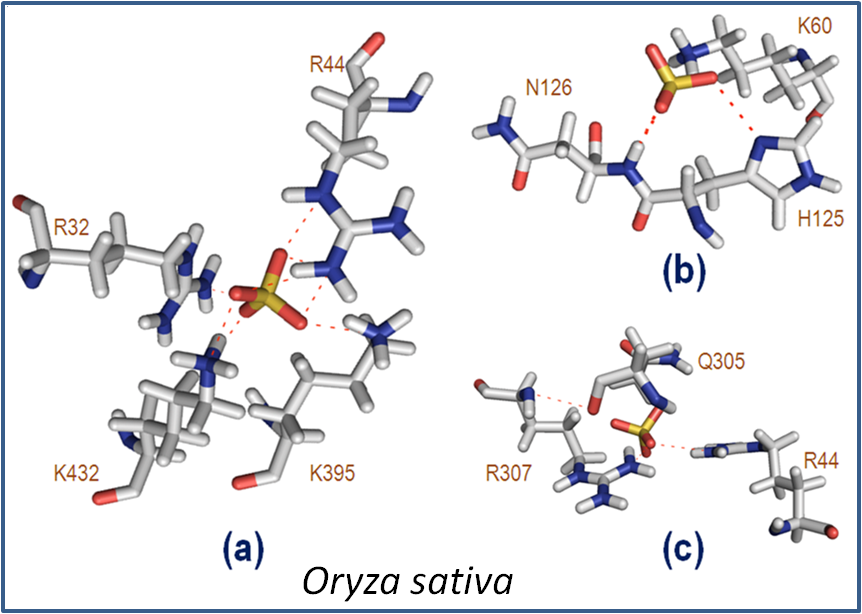

Supplement: Supplementary file 1 — Supplementary Figure S1. Ramachandran plot of both monocot and dicot AGPase small subunit. (i) Oryza sativa ssp. japonica AGPase small subunit, (ii) Hordeum vulgare AGPase small subunit, (iii) Triticum aestivum AGPase small subunit, (iv) Arabidopsis thaliana AGPase small subunit, (v) Solanum lycopersicum AGPase small subunit, (vi) Beta vulgaris AGPase small subunit, (vii) Vicia faba1 AGPase small subunit, (viii) Vicia faba2 AGPase small subunit, (ix) Cicer arietinum1 AGPase small subunit, (x) Cicer arietinum2 AGPase small subunit, (xi) Brassica napus AGPase small subunit . Supplementary Figure S2. Theoritical three dimensional models of both monocot and dicot AGPase small subunit. (i) Oryza sativa ssp. japonica AGPase small subunit, (ii) Hordeum vulgare AGPase small subunit, (iii) Triticum aestivum AGPase small subunit, (iv) Arabidopsis thaliana AGPase small subunit, (v) Solanum lycopersicum AGPase small subunit, (vi) Beta vulgaris AGPase small subunit, (vii) Vicia faba1 AGPase small subunit, (viii) Vicia faba2 AGPase small subunit, (ix) Cicer arietinum1 AGPase small subunit, (x) Cicer arietinum2 AGPase small subunit, (xi) Brassica napus AGPase small subunit [file 583606.f1.zip › supplementary/O. sativa.TIF]

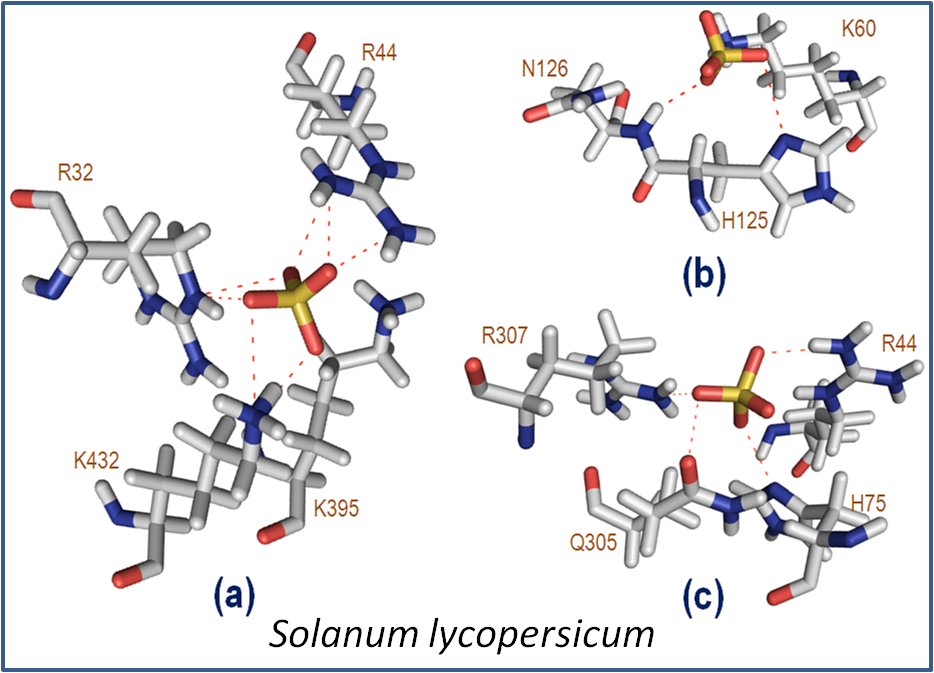

Supplement: Supplementary file 1 — Supplementary Figure S1. Ramachandran plot of both monocot and dicot AGPase small subunit. (i) Oryza sativa ssp. japonica AGPase small subunit, (ii) Hordeum vulgare AGPase small subunit, (iii) Triticum aestivum AGPase small subunit, (iv) Arabidopsis thaliana AGPase small subunit, (v) Solanum lycopersicum AGPase small subunit, (vi) Beta vulgaris AGPase small subunit, (vii) Vicia faba1 AGPase small subunit, (viii) Vicia faba2 AGPase small subunit, (ix) Cicer arietinum1 AGPase small subunit, (x) Cicer arietinum2 AGPase small subunit, (xi) Brassica napus AGPase small subunit . Supplementary Figure S2. Theoritical three dimensional models of both monocot and dicot AGPase small subunit. (i) Oryza sativa ssp. japonica AGPase small subunit, (ii) Hordeum vulgare AGPase small subunit, (iii) Triticum aestivum AGPase small subunit, (iv) Arabidopsis thaliana AGPase small subunit, (v) Solanum lycopersicum AGPase small subunit, (vi) Beta vulgaris AGPase small subunit, (vii) Vicia faba1 AGPase small subunit, (viii) Vicia faba2 AGPase small subunit, (ix) Cicer arietinum1 AGPase small subunit, (x) Cicer arietinum2 AGPase small subunit, (xi) Brassica napus AGPase small subunit [file 583606.f1.zip › supplementary/S. lycopersicum.TIF]

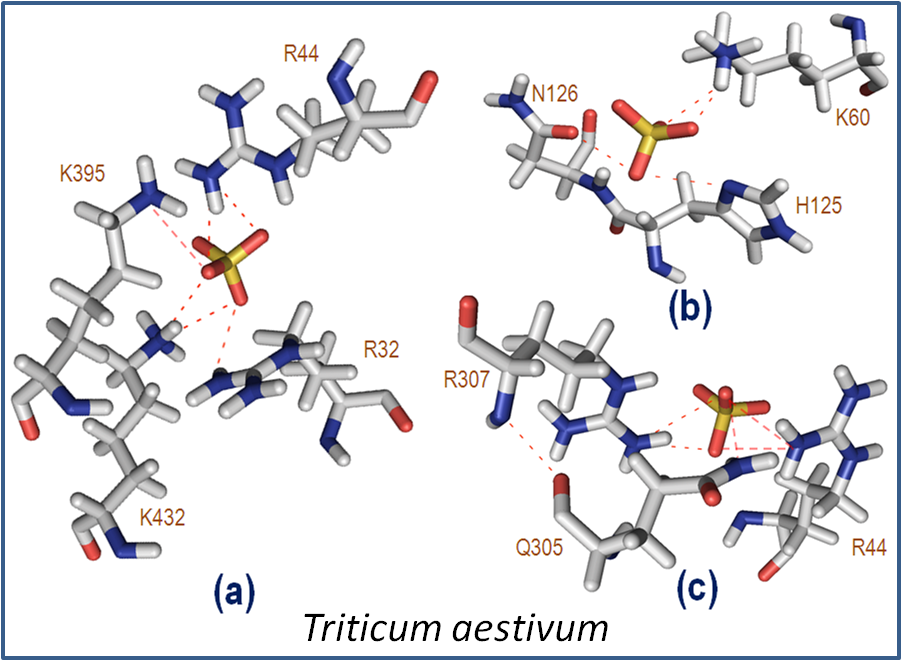

Supplement: Supplementary file 1 — Supplementary Figure S1. Ramachandran plot of both monocot and dicot AGPase small subunit. (i) Oryza sativa ssp. japonica AGPase small subunit, (ii) Hordeum vulgare AGPase small subunit, (iii) Triticum aestivum AGPase small subunit, (iv) Arabidopsis thaliana AGPase small subunit, (v) Solanum lycopersicum AGPase small subunit, (vi) Beta vulgaris AGPase small subunit, (vii) Vicia faba1 AGPase small subunit, (viii) Vicia faba2 AGPase small subunit, (ix) Cicer arietinum1 AGPase small subunit, (x) Cicer arietinum2 AGPase small subunit, (xi) Brassica napus AGPase small subunit . Supplementary Figure S2. Theoritical three dimensional models of both monocot and dicot AGPase small subunit. (i) Oryza sativa ssp. japonica AGPase small subunit, (ii) Hordeum vulgare AGPase small subunit, (iii) Triticum aestivum AGPase small subunit, (iv) Arabidopsis thaliana AGPase small subunit, (v) Solanum lycopersicum AGPase small subunit, (vi) Beta vulgaris AGPase small subunit, (vii) Vicia faba1 AGPase small subunit, (viii) Vicia faba2 AGPase small subunit, (ix) Cicer arietinum1 AGPase small subunit, (x) Cicer arietinum2 AGPase small subunit, (xi) Brassica napus AGPase small subunit [file 583606.f1.zip › supplementary/T. aestivum.TIF]

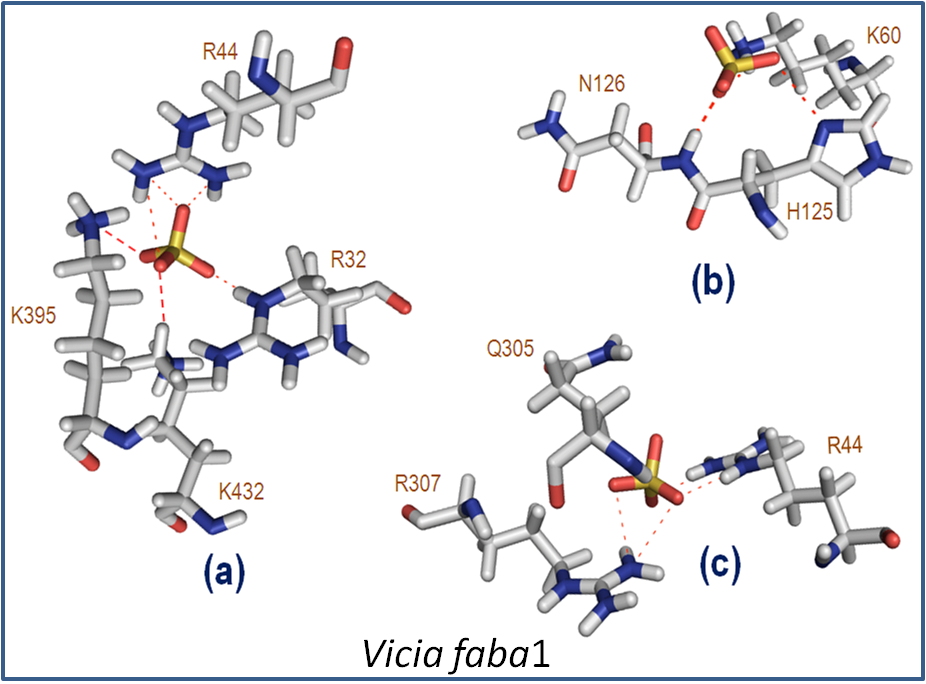

Supplement: Supplementary file 1 — Supplementary Figure S1. Ramachandran plot of both monocot and dicot AGPase small subunit. (i) Oryza sativa ssp. japonica AGPase small subunit, (ii) Hordeum vulgare AGPase small subunit, (iii) Triticum aestivum AGPase small subunit, (iv) Arabidopsis thaliana AGPase small subunit, (v) Solanum lycopersicum AGPase small subunit, (vi) Beta vulgaris AGPase small subunit, (vii) Vicia faba1 AGPase small subunit, (viii) Vicia faba2 AGPase small subunit, (ix) Cicer arietinum1 AGPase small subunit, (x) Cicer arietinum2 AGPase small subunit, (xi) Brassica napus AGPase small subunit . Supplementary Figure S2. Theoritical three dimensional models of both monocot and dicot AGPase small subunit. (i) Oryza sativa ssp. japonica AGPase small subunit, (ii) Hordeum vulgare AGPase small subunit, (iii) Triticum aestivum AGPase small subunit, (iv) Arabidopsis thaliana AGPase small subunit, (v) Solanum lycopersicum AGPase small subunit, (vi) Beta vulgaris AGPase small subunit, (vii) Vicia faba1 AGPase small subunit, (viii) Vicia faba2 AGPase small subunit, (ix) Cicer arietinum1 AGPase small subunit, (x) Cicer arietinum2 AGPase small subunit, (xi) Brassica napus AGPase small subunit [file 583606.f1.zip › supplementary/V. faba1.TIF]

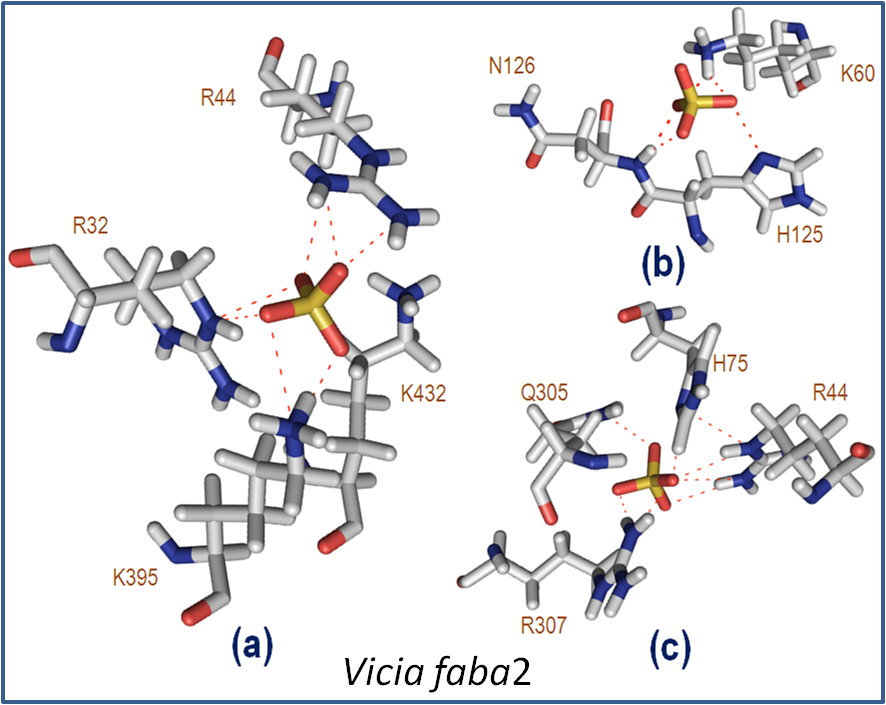

Supplement: Supplementary file 1 — Supplementary Figure S1. Ramachandran plot of both monocot and dicot AGPase small subunit. (i) Oryza sativa ssp. japonica AGPase small subunit, (ii) Hordeum vulgare AGPase small subunit, (iii) Triticum aestivum AGPase small subunit, (iv) Arabidopsis thaliana AGPase small subunit, (v) Solanum lycopersicum AGPase small subunit, (vi) Beta vulgaris AGPase small subunit, (vii) Vicia faba1 AGPase small subunit, (viii) Vicia faba2 AGPase small subunit, (ix) Cicer arietinum1 AGPase small subunit, (x) Cicer arietinum2 AGPase small subunit, (xi) Brassica napus AGPase small subunit . Supplementary Figure S2. Theoritical three dimensional models of both monocot and dicot AGPase small subunit. (i) Oryza sativa ssp. japonica AGPase small subunit, (ii) Hordeum vulgare AGPase small subunit, (iii) Triticum aestivum AGPase small subunit, (iv) Arabidopsis thaliana AGPase small subunit, (v) Solanum lycopersicum AGPase small subunit, (vi) Beta vulgaris AGPase small subunit, (vii) Vicia faba1 AGPase small subunit, (viii) Vicia faba2 AGPase small subunit, (ix) Cicer arietinum1 AGPase small subunit, (x) Cicer arietinum2 AGPase small subunit, (xi) Brassica napus AGPase small subunit [file 583606.f1.zip › supplementary/V. faba2.TIF]
